# The neural and cognitive basis of expository text comprehension

**DOI:** 10.1038/s41539-024-00232-y

**Published:** 2024-03-21

**Authors:** Timothy A. Keller, Robert A. Mason, Aliza E. Legg, Marcel Adam Just

**Affiliations:** Center for Cognitive Brain Imaging, Department of Psychology, Carnegie Mellon University, Pittsburgh, PA 15213 USA

**Keywords:** Language, Human behaviour, Cognitive neuroscience

## Abstract

As science and technology rapidly progress, it becomes increasingly important to understand how individuals comprehend expository technical texts that explain these advances. This study examined differences in individual readers’ technical comprehension performance and differences among texts, using functional brain imaging to measure regional brain activity while students read passages on technical topics and then took a comprehension test. Better comprehension of the technical passages was related to higher activation in regions of the left inferior frontal gyrus, left superior parietal lobe, bilateral dorsolateral prefrontal cortex, and bilateral hippocampus. These areas are associated with the construction of a mental model of the passage and with the integration of new and prior knowledge in memory. Poorer comprehension of the passages was related to greater activation of the ventromedial prefrontal cortex and the precuneus, areas involved in autobiographical and episodic memory retrieval. More comprehensible passages elicited more brain activation associated with establishing links among different types of information in the text and activation associated with establishing conceptual coherence within the text representation. These findings converge with previous behavioral research in their implications for teaching technical learners to become better comprehenders and for improving the structure of instructional texts, to facilitate scientific and technological comprehension.

## Introduction

Learning technical information from text in a technological world is a central cognitive skill that is taught in schools, tested by educational institutions and industry employers, and remains a crucial ability for a growing number of occupations as well as for everyday life. Reading comprehension skills are engaged every time we read a user’s manual, a Wikipedia article, or a technical handbook. Neurocognitive research approaches using functional brain imaging have the potential of substantially increasing the understanding of the processes underlying technical reading comprehension, and concomitantly, revealing possible opportunities for enhancing this process.

This study used brain imaging to address two central sets of questions regarding the processes necessary for comprehending technical information from a text. First, is it possible to identify the neuropsychological processes that distinguish good and poor comprehenders? In what way are some people better able than others to grasp from their reading how a technological device works? What are they doing differently than poor comprehenders? Although there is a long history of psychological and educational research approaches to these questions^1–6^, as described below, there is much less converging evidence from neuroscience research. The answers to these questions derived from brain imaging may converge with behavioral research in suggesting comprehension strategies that can be taught to learners to enhance their comprehension of novel technical material. It also may add new insight into the underlying processes used by better comprehenders of technical material, suggesting additional comprehension strategies.

Second, what conceptual and structural aspects of technical texts are associated with better or poorer comprehension? Are there certain properties shared by more comprehensible texts that facilitate effective comprehension? Do the processes used to successfully acquire and integrate new knowledge reveal how writers should compose and structure a technical passage to foster comprehension and memory for novel technical material? Again, previous behavioral research has addressed these questions^5,7–10^, but far less is known about how the brain responds to the properties of technical texts. Exploring the answers to these questions from a neurocognitive approach may provide additional insight into how texts can be better engineered to improve the comprehensibility and retention of new technical information.

The present study addressed these questions by acquiring brain activation data (fMRI) as participants, varying in comprehension skills, read short technical expository passages for comprehension. The choice of materials and experimental design was intended to make the task ecologically valid, resembling a real-world comprehension task, with the goal of enhancing the understanding of how new technical knowledge is acquired from text in the context of a realistic comprehension situation. The fMRI data were acquired during the reading of the passages and the goal of the study was to develop an understanding of how the neurocognitive processes that occur during reading are related to comprehending the text, as assessed by a multiple-choice comprehension test. The comprehension of individual participants and the comprehensibility of individual technical passages were used to identify brain areas whose activity underlies comprehension performance.

There is increasing recognition that focusing on brain localization of individual component processes of reading comprehension may fail to explain the complexity of real-world text comprehension^11^, and similar concerns have been raised about purely behavioral studies of expository text comprehension^12,13^. Many previous findings are based on experimental studies designed to isolate a single linguistic process while controlling the effects of others^14,15^. This type of functional decomposition may obscure the effects of other processes that can make comprehension the outcome of a more dynamic and interactive network of neural processing. This concern becomes especially salient when considering individual differences in comprehension performance. Recent neuroimaging studies of text comprehension have used naturalistic designs that attempt to investigate the collective neural signature of the participating processes^16–18^. The present study used realistic technical passages to be learned in a conventional reading comprehension task.

Several behavioral studies have investigated the specific processes that are engaged by the distinguishing properties of technical texts^6,9,13,19^. Expository technical texts typically describe a set of related concepts and their co-functioning in various events^20,21^. Compared to narrative texts, technical texts often contain less familiar vocabulary, are less likely to reflect everyday experiences, and use more complex linguistic structures, properties that can make them more difficult to comprehend and learn^4^. In addition, technical texts typically require fewer inferences to be made^19^, and their comprehension may depend more heavily on previous knowledge^22^. Only a few previous neuroimaging studies have investigated the neural basis of expository text comprehension^23–28^, despite its relevance for STEM education and job performance. The present study addresses this gap between behavioral and neuroimaging research in understanding expository text comprehension.

Reading comprehension depends on a large number of component processes, and the efficiency with which each one of them is executed can vary across individual readers. At the single-word and single-sentence levels, these processes include decoding of word forms, retrieval and selection of word meanings, syntactic processing of multi-word constituents, construction of propositions and assignment of case (or pragmatic) roles, establishment of cohesion among propositions, and the maintenance of the propositions in working memory. At the discourse level, additional processes are engaged, including the establishment of global coherence across sentences, maintenance of distantly related propositions in working memory, retrieval of semantic knowledge structures from long-term memory, and the integration of retrieved knowledge with information from the text. Particularly relevant for technical comprehension is the construction of a mental model or situation model of the referents of the text and their interrelations^2,29^. The mental model is constructed with the aid of the reader’s existing world knowledge to enable various types of inferences (e.g., bridging, predictive, causal, elaborative) that complete the unspoken parts of the text^6^.

Individual comprehenders may differ in their proficiency in any of these processes^30–33^. In addition, they may differ in the background knowledge they bring to the comprehension task, and as one would expect, prior knowledge in a domain affects how proficiently new knowledge in that domain is acquired^34,35^. Furthermore, proficiency in the underlying processes interacts with both prior knowledge and the structure of the text in determining comprehension performance^7,8,36^. The present study assessed which of the underlying processes, identified in regional patterns of brain activation, accounted for differences among individuals in their ultimate reading comprehension performance.

Neuroimaging studies have indicated a set of underlying brain regions that become activated by the engagement of text comprehension processes. Lexical retrieval and semantic knowledge representation involve an extensive network including the left-lateralized parietal, temporal, and inferior frontal lobe areas that make up the core language network, and they also involve bilateral activity in the ventral temporal cortex, the anterior temporal lobes, the hippocampus, and the ventromedial and ventrolateral prefrontal cortex^37–39^. Reading comprehension of extended discourse also depends on a brain network that carries out conceptual and semantic integration, which overlaps with semantic processing and representation regions and also includes lateral areas of the prefrontal cortex in both hemispheres^40,41^.

The construction of a mental model includes using previous knowledge drawn from semantic memory (knowledge of words, facts, and concepts not associated with a specific learning experience) and episodic or autobiographical memory (knowledge of past experiences). Semantic memory storage is distributed across the neocortex in a number of brain regions specialized for particular types of information^17,39,42,43^, and the retrieval of semantic knowledge and its integration with new or inconsistent prior knowledge is associated with lateral prefrontal regions^44,45^. Episodic memory storage is associated with the medial temporal lobe and hippocampus^46^ and episodic retrieval is associated with medial prefrontal regions^47–49^.

In sum, there is extensive literature associating individual brain regions and networks of regions with particular text comprehension processes. This literature provides the basis for inferring which processes are occurring given the observed brain activity during the reading of a particular text by a particular participant. These associations are based on a large literature of multiple studies in which there is activation in particular regions during the execution of a task assumed to engage a given process. This literature, such as the compendium provided by the Neurosynth database^50^ can be leveraged to clarify which processes are occurring by providing posterior probabilities of the likelihood that a process is associated with activation in a particular area^51,52^. In our study, the degree of engagement of a given process (assessed by its associated activation level in a given region) during the reading of a technical passage was used to predict the degree of mastery of the passage content.

In this study, the fMRI brain activity of 31 college-age participants of varying comprehension skills was acquired while they read a set of 16 technical passages for comprehension three times, followed by a multiple-choice comprehension test. Behavioral research has demonstrated that repetition of spoken passages increases memory performance, and also results in qualitative shifts in processing^5,53,54^, and brain imaging studies have indicated that there are corresponding related changes in regional brain activity over repeated hearing or reading of the same passage^25,55^.

The brain activation observed during the reading was used to predict how well individual participants would perform on the comprehension test and to infer which of the underlying processes distinguished the better comprehenders from the poorer comprehenders. (We refer to the prediction of comprehension performance from brain activity in the statistical sense because a direct causal link from neural activity to cognitive performance is not attainable in fMRI studies.) Similarly, brain activation during reading was used to indicate which passages would be more difficult to comprehend than others and to infer which of the underlying processes were modulated by passage difficulty.

## Results

### Summary

The brain activation patterns acquired during participants’ reading of the technical passages were systematically predictive of two types of outcomes.

First, regional brain activation during the reading of the technical passages was related to performance on a post-scan comprehension test. The brain regions whose activation best predicted comprehension of the technical content of the passages were strongly associated with several main types of processes: verbal working memory supporting syntactic and semantic language comprehension, retrieval of prior knowledge, spatial aspects of mental model construction, and building new semantic knowledge structures.

A second finding was that the regional brain activation during the reading of the technical passages was also related to how easy or hard the passage was to comprehend. The modulation of passage comprehension difficulty was related to the activation in brain regions associated with establishing links among different types of knowledge representations; typically these different types of knowledge representations are distributed across the cortex. The modulation was also related to activation in regions associated with establishing conceptual coherence.

### Brain regions and processes related to individual participant’s comprehension

**Fig. 1:**
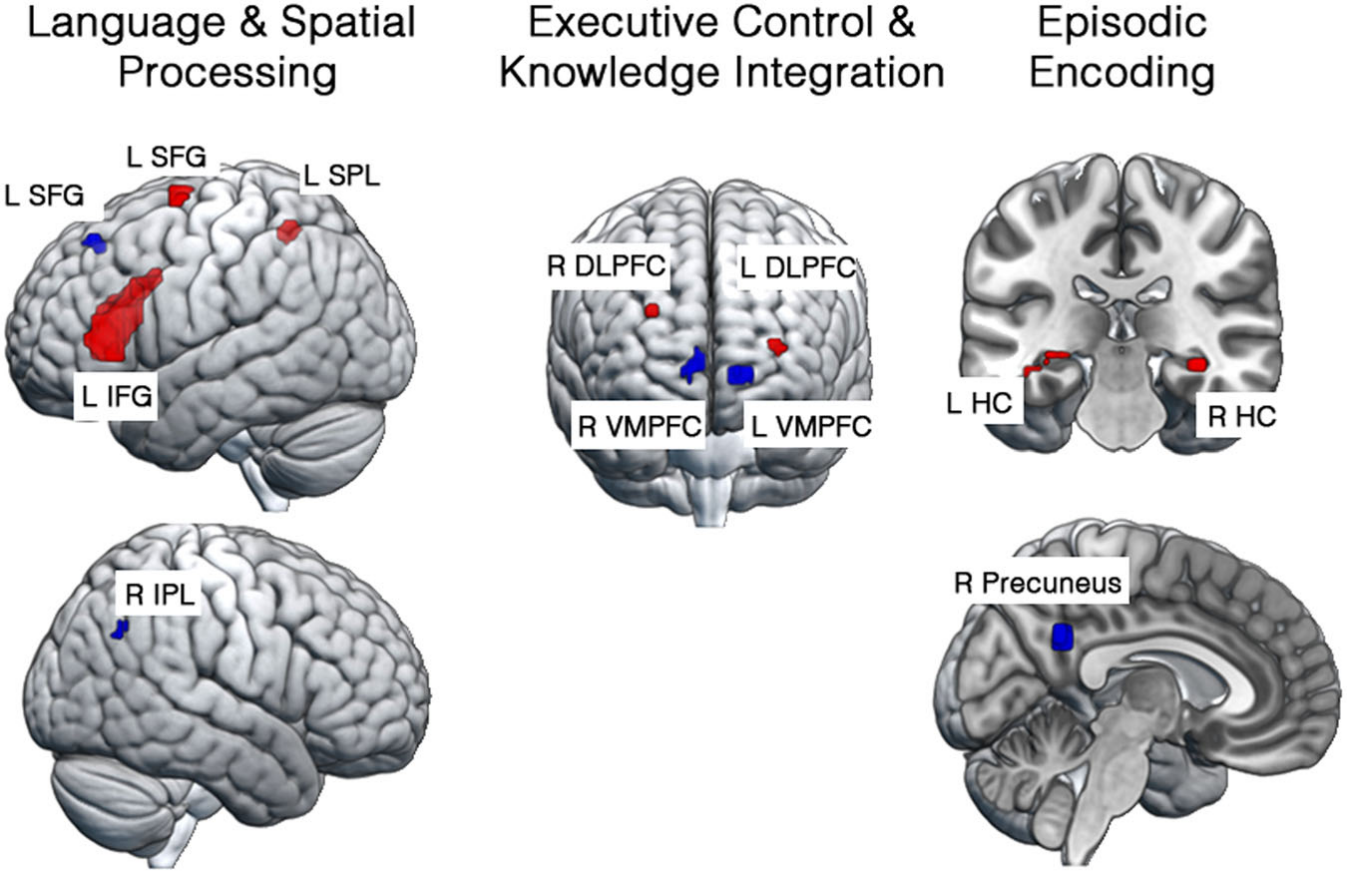
Regions where brain activation was related to individual differences in reading comprehension. Red: Regions where activation was greater for participants who were better comprehenders. Blue: Regions where activation was greater for participants who were poorer comprehenders. See Table 1 for region abbreviations.

A set of regions associated with language processing and memory, spatial imagery, episodic encoding, and semantic knowledge retrieval and reorganization was more highly activated in readers who displayed better comprehension of the technical passages. Positive correlations were found between technical text comprehension and activation in language processing areas (the *pars opercularis* and *pars triangularis portions* of the left inferior frontal gyrus (L IFG)); in spatial processing areas (the left superior parietal lobule (SPL)); in a semantic integration area (left dorsolateral prefrontal cortex (L DLPFC); and in areas involved in the encoding and consolidation of new declarative knowledge (left and right hippocampal areas). The areas showing a positive relationship between activation and comprehension of the passages are shown in red in Fig. 1. The peak *t*-values and MNI coordinates for clusters of voxels showing a difference in correlation with comprehension from zero and MNI coordinates are presented in Table 1.

**Table 1:** Regions of interest based on their correlation between activation during reading and comprehension

Regions of Interest	Abbreviation in Figure 1	Peak *t*-value	Peak MNI coordinates			Volume (mm^3^)	Processes
			x	y	z		
Regions with a Positive Correlation Between Activation and Comprehension							
*Left Inferior Frontal	L IFG	3.36	−39	26	13	9,099	Language Processing
Left Superior Frontal	L SFG	2.40	−24	−1	73	540	Premotor Processing
Left Superior Parietal	L SPL	2.24	−24	−49	55	621	Spatial Processing
Left Dorsolateral Prefrontal	L DLPFC	1.90	−30	47	7	162	Semantic Knowledge Integration
*Right Dorsolateral Prefrontal	R DLPFC	2.10	24	29	25	216	Semantic Knowledge Integration
Left Hippocampus	L HC	2.09	−36	−40	−5	594	Episodic Encoding
*Right Hippocampus	R HC	2.17	33	−31	11	135	Episodic Encoding
Regions with a Negative Correlation Between Activation and Comprehension							
Right Ventromedial Prefrontal	R VMPFC	2.70	6	68	1	436	Episodic Knowledge Integration
*Left Ventromedial Prefrontal	L VMPFC	2.64	−12	68	−5	459	Episodic Knowledge Integration
Left Superior Frontal	L SFG	2.09	−24	38	52	216	Episodic Retrieval
Right Precuneus	R Precuneus	2.47	18	−52	28	918	Episodic Retrieval
Right Inferior Parietal	R IPL	2.04	60	−58	37	135	Coherence Detection

Entries with an * are the most predictive variables of individual comprehension performance.

Other brain areas showed a negative relation between comprehension of the technical passages and activation during the reading of the passages (greater activation was associated with poorer comprehending of participants). These areas are associated with the retrieval of information from episodic or autobiographical memory (left superior frontal gyrus (L SFG), left and right ventromedial prefrontal cortex (L and R VMPFC), and the right precuneus (R Precuneus)), and areas associated with the integration of newly learned knowledge with information retrieved from episodic or autobiographical memory (left and right medial prefrontal cortex). These areas are shown in blue in Fig. 1 and are presented at the bottom of Table 1.

The activation in regions showing a positive or negative relationship between activation and comprehension performance was remarkably similar across the three presentations. Regions positively related to comprehension in all three readings of the sentences included the left inferior frontal gyrus (L IFG), the left superior parietal lobule (L SPL), the left middle frontal gyrus (L MFG), and areas involved in the encoding and consolidation of new declarative knowledge (left and right hippocampal areas and right precuneus). Supplementary Fig. 1 in the online Supplementary Information shows the regions related to comprehension separately for each presentation.

### Multiple regression modeling of the relation between activation and individual participant’s comprehension performance

**Fig. 2:**
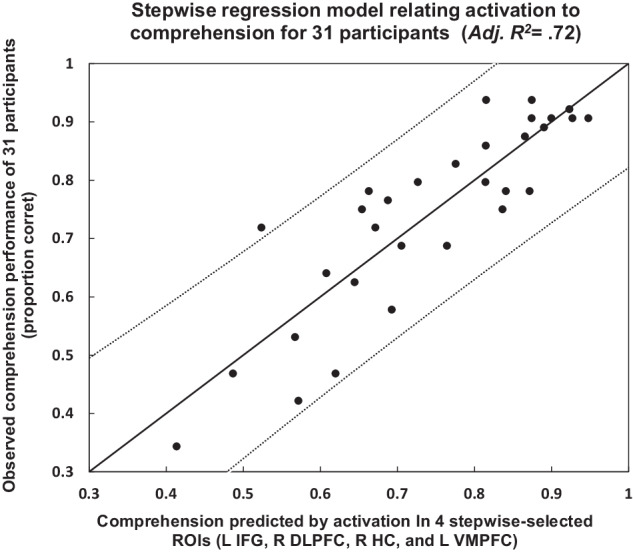
Relationship between individual participant comprehension performance and performance predicted from activation in key cortical regions. Each point represents a participant. Values on the axes are the proportion of correct responses on a multiple-choice comprehension test. Dotted lines show the 95% confidence intervals on prediction.

A stepwise regression indicated that activation levels in four regions were particularly related to individual participants’ comprehension performance: left ventromedial prefrontal cortex (L VMPFC), left inferior frontal gyrus (L IFG), right dorsolateral prefrontal cortex (R DLPFC), and the right hippocampus (R HC). These were also the most influential regions as measured by their standardized regression weights (*ß*s) in a model including all regions as predictors. The brain activation levels of each participant (averaged over the three readings of the passage) in each of the 12 regions described above were entered as the independent variables in the stepwise regression model to evaluate their relationship to the mean comprehension performance of each participant (averaged over the 16 passages). The selected regions were the first four independent variables that entered the model (thus, a forward selection process, with the minimum Schwartz Bayesian Criterion dictating when to stop adding variables). The resulting model was free from serious multicollinearity (variance inflation < 1.5 for each of the selected variables, with the minimum tolerance among them being 0.74). As the direction of correlations shown in Fig. 1 and in Table 1 suggest, only the left ventromedial prefrontal cortex (L VMPFC) had a negative regression weight. Interestingly, it was also the most influential of the four variables selected by the stepwise procedure (*ß* = −0.49); the negative regression weight indicates that participants who performed more poorly on the comprehension test tended to have higher activation in this area. The remaining three predictive regions had positive regression weights. The better comprehenders had higher levels of activity in L IFG, R DLPFC, and R HC. The fit of this model using activation in these four regions as independent variables was reliable (*F*(4,26) = 20.43, MSE = 0.007, *p* < 0.00001) and resulted in an *R*^2^ of .76 (adjusted *R*^2^ = 0.72, Fig. 2).

**Fig. 3:**
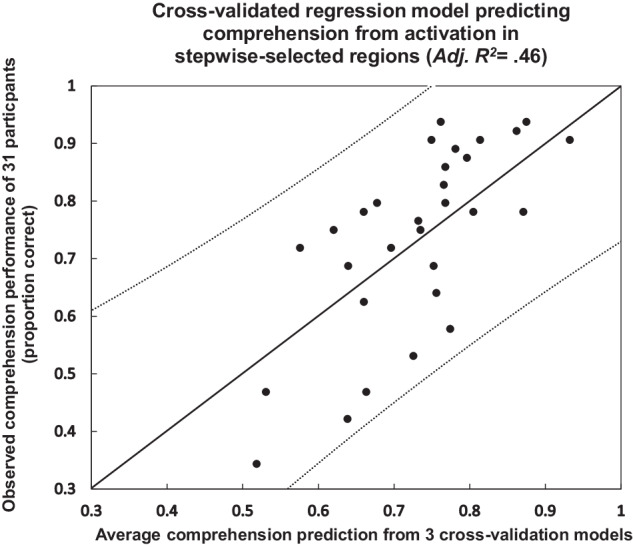
Cross-validated relationship between individual participant comprehension performance and activation in key cortical regions (L IFG, R DLPFC, L VMPFC, R HC). Each point represents a participant. Values on the axes are the proportion of correct responses on a multiple-choice comprehension test. Dotted lines show the 95% confidence intervals on prediction.

Cross-validation methods that trained the models on a subset of the data and tested them on the unseen subset established good predictive generalizability of the model. The results of the stepwise analysis reported above indicated that individual participant comprehension performance was reliably associated with brain activation in the selected regions from the full dataset including all three readings of the sentences. This cross-validation procedure establishes predictive validity across independent datasets, beyond showing predictiveness across correlated datasets, as the full stepwise model above does. Cross-validation was carried out by using the data from two presentations (averaged over two readings of the full set of passages) to select ROIs and train a regression model. Comprehension performance in the third, left-out presentation was then predicted using the regression weights from the training model. This cross-validation was repeated using each pair of presentations for training and each left-out presentation for testing. The ROIs selected for training in each cross-validation fold were again based on the correlation of activation and comprehension but were calculated separately for each pair of training presentations. Each of these cross-validated models demonstrated a reliable relationship between activation in the selected ROIs and comprehension performance (*R*^2^ = 0.47, (*F*(4,26) = 5.67, MSE = 0.017, *p* = 0.00203), *R*^2^ = 0.49 (*F*(4,26) = 6.18, MSE = 0.016, *p* = 0.00123), and *R*^2^ = 0.49 (*F*(4,26) = 6.23, MSE = 0.016, *p* = 0.00114), for the three possible leave-one-out folds). The fit of the average of the three cross-validation models is presented in Fig. 3.

### Brain regions and processes related to individual passage comprehension difficulty

**Fig. 4:**
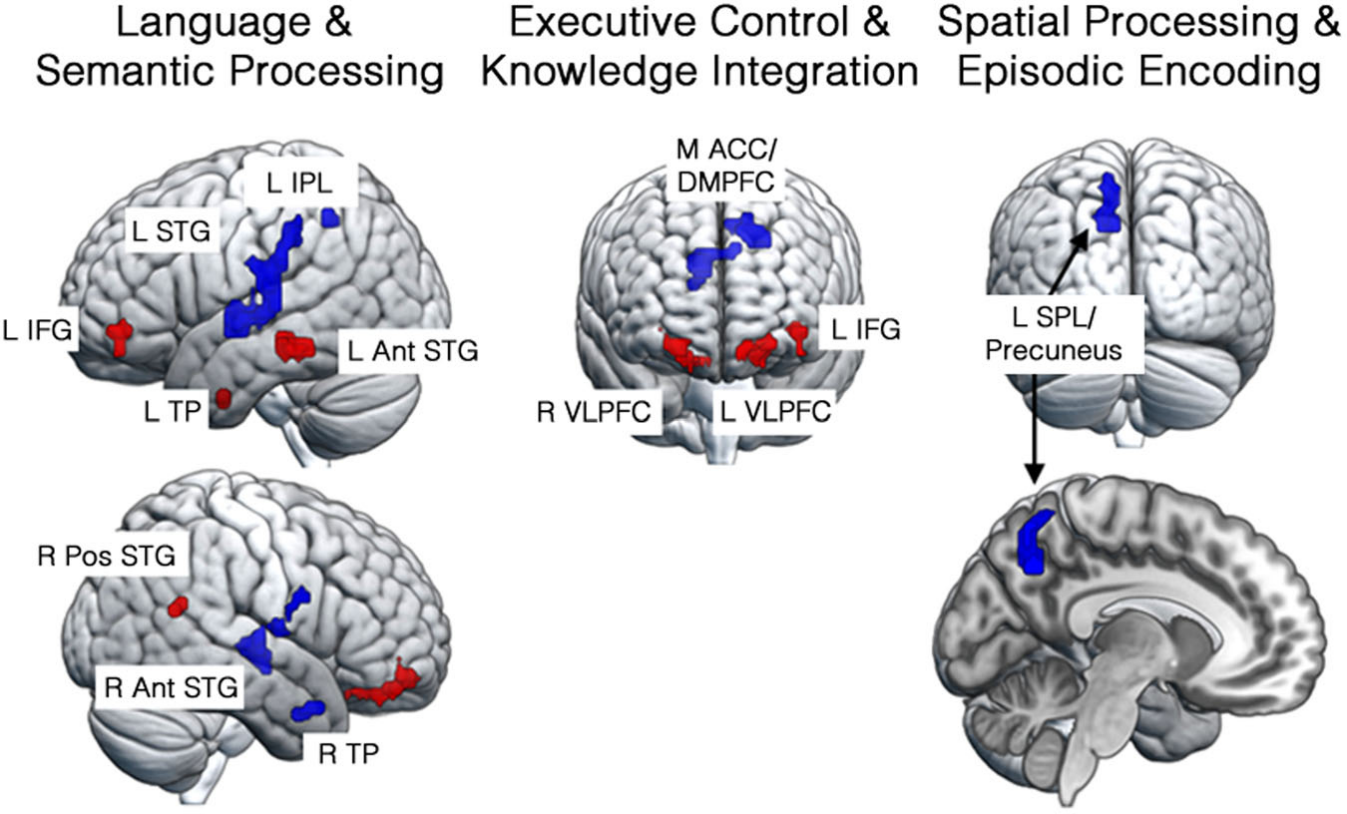
Regions of correlation between brain activation during the reading of individual passages and comprehension performance on the corresponding passages. Red: Regions whose activation was greater for better-comprehended passages. Blue: Regions whose activation was greater for more poorly comprehended passages. See Table 2 for region abbreviations.

The comprehensibility of each of the 16 passages was related to the levels of brain activity in 13 brain regions whose voxelwise activation was reliably (*p* < 0.05) correlated with the mean comprehension level (*t*-tests comparing the values to zero on these voxelwise maps of the correlation coefficients for the 31 participants identified in these 13 regions, as shown in Fig. 4 and Table 2).

**Table 2:** Regions of interest based on activity correlations with passage comprehensibility during reading

Region of Interest	Abbreviation in Figure 3	Peak *t*-value	Peak MNI coordinates			Volume (mm^3^)	Processes
			x	y	z		
Positively related to passage comprehensibility							
*Left Inferior Frontal (*pars opercularis*)	L IFG	2.04	45	50	−8	810	Semantic Processing
Left Temporal Pole	L TP	2.58	−39	−1	−38	351	Semantic Processing
Left Middle Temporal	L MTG	2.98	−57	−34	−17	1998	Semantic Processing
Left Ventrolateral Prefrontal	L VLPFC	2.48	−21	47	−14	1836	Semantic Knowledge Integration
Right Ventrolateral Prefrontal	R VLPFC	2.35	21	53	−14	1674	Semantic Knowledge Integration
Right Post. Sup. Temporal	R PosSTG	2.46	45	−52	19	432	Semantic Processing
Negatively related to passage comprehensibility							
Left Superior Temporal	L STG	2.70	−51	−22	1	6048	Semantic Processing
Left Parietal/Precuneus	L SPL/PC	3.05	−12	−61	43	2214	Spatial Visualization
*Left Inferior Parietal	L IPL	2.23	42	−49	19	405	Semantic processing
Left Dorsomedial Prefrontal	L DMPFC	2.68	−12	20	46	1863	Executive Control
*Medial Anterior Cingulate/Dorsomedial PFC	M ACG/D MPFC	4.08	12	14	28	2943	Executive Control
*Right Temporal Pole	R TP	2.58	42	11	−26	675	Semantic Processing
Right Superior Temporal	R AntSTG	2.06	57	−10	−2	2592	Semantic Processing

Entries with an * are the most predictive variables of individual comprehension performance.

**Fig. 5:**
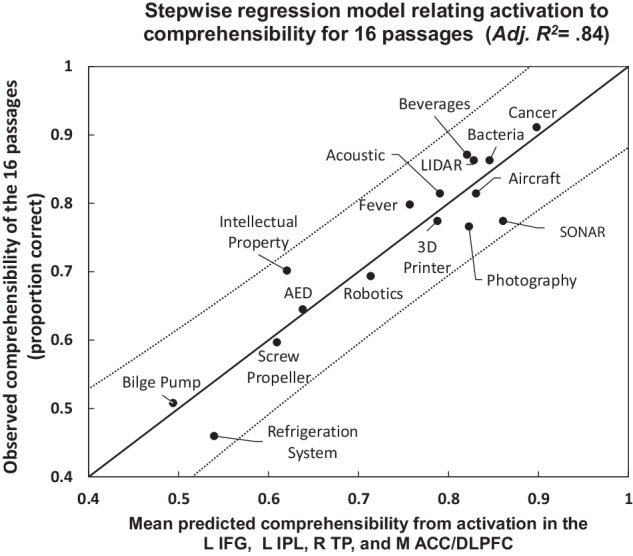
Relationship between individual passage comprehensibility and activation in key cortical regions. Each point represents a passage. Values on the axes are the proportion of correct responses on a multiple-choice comprehension test. Dotted lines show the 95% confidence intervals on prediction. See the “Methods” section for the full passage titles.

The activation levels in these 13 brain regions were entered as predictor variables in a stepwise regression model that identified four regions that best explained the variance in passage comprehensibility based on the minimum Schwartz Bayesian Criterion: the *pars opercularis* of the left inferior frontal gyrus (L IFG), the right temporal pole (R TP), the left inferior parietal lobule, and the medial anterior cingulate/dorsomedial prefrontal cortex (M ACC/DMPFC). This reduced model, based on the regions whose activation was correlated with comprehension, straightforwardly predicted the mean comprehension of each passage (*R*^2^ = 0.88 (Adj. *R*^2^ = 0.84), *F*(4,11) = 20.02, MSE = 0.003, *p* = 0.00005), as shown in Fig. 5. (The cross-validated model with predictive validity is reported below). The four selected variables entered the stepwise model and stayed without removal (a forward regression model). Two of the four selected regions were also the most influential as measured by their standardized regression weights in the full 13-ROI model (the M ACC/DMPFC *ß* = −0.99, L IFG *ß* = 0.74), and there were no collinearity problems with the four selected predictors (all variance inflation estimates < 2.0, all tolerances > 0.5). These regions predictive of the difficulty of comprehending individual passages are involved in lexical access and serve as a semantic hub that binds together semantic knowledge of different types^37,38,56,57^.

The ability to predict comprehension difficulty at the passage level from activation was cross-validated using the data from each pair of presentations (readings of the set of passages) to select ROIs and to train a regression model. As with the prediction of comprehension at the participant level reported above, data from the remaining presentation was used to test the generalizability of the model. (The presentation-specific regions of activity are shown in Supplementary Fig. 2.) Each of these cross-validation models demonstrated a reliable relationship between activation in the selected ROIs and comprehensibility (*R*^2^ = 0.79, (*F*(4,11) = 10.37, MSE = 0.005, *p* = 0.00099), *R*^2^ = 0.73 (*F*(4,11) = 7.60, MSE = 0.006, *p* = 0.00344), and *R*^2^ = 0.76 (*F*(4,11) = 8.57, MSE = 0.007, *p* = 0.00215), for the three possible leave-one-out folds). The mean predicted comprehensibility of each passage across the three training/test combinations resulted in an *R*^2^ of 0.44 (*F*(1,14) = 11.08, MSE = 0.01, *p* = 0.004970) (see Supplementary Fig. 3 for a plot of the cross-validated model fit.).

### Predicting individual differences in comprehension on the basis of psychometrically assessed cognitive ability

An interesting question is whether the brain activity measures were superior to behavioral measures of cognitive ability in predicting individuals’ reading comprehension performance. (Supplementary Table 1 presents the correlations among all individual differences measures.) The Nelson–Denny Reading Comprehension measure^58^, which is very similar to the target comprehension test and hence is not an independent predictor, was unsurprisingly significantly correlated with technical passage comprehension (*R*^2^ = 0.40, *F*(1,29) = 19.37, MSE = 0.017, *p* = 0.000133). Other psychometric measures were also mildly predictive of comprehension performance. The Raven Progressive Matrices Test^59^, a measure of fluid intelligence, was reliably related to comprehension (*R*^2^ = 0.30, *F*(1,29) = 12.18, MSE = 0.022, *p* = 0.001565). The Reading Span Test^60^, a measure of working memory capacity for language comprehension, was also reliably related (*R*^2^ = 0.22, *F*(1,29) = 8.39, MSE = 0.022, *p* = 0.00711). Also mildly related was the Bennett Mechanical Comprehension Test^61^, a measure of mechanical aptitude testing spatial visualization and knowledge of basic physical and mechanical laws and their application in solving mechanical problems (*R*^*2*^ = 0.18, *F*(1,29) = 6.50, MSE = 0.023, *p* = 0.016336). A multiple regression model using the latter three psychometric measures to predict comprehension resulted in a multiple *R*^2^ of 0.36 (*F*(3,27) = 4.96, MSE = 0.019, *p* = 0.00188). By comparison, as reported above, the brain activation measures in four brain regions (L IFG, L IPL, M ACC/DLPFC, and R HC) predicted comprehension with a multiple *R*^2^ of 0.76. To quantitatively compare which type of measure was more predictive of comprehension, one set of measures (psychometric or brain imaging) was included in an initial model and the alternative set of measures was added to that model to test whether the improvement in prediction was reliable. The resulting analysis indicated that the addition of the psychometric measures to the brain imaging measures did not result in a significant improvement in prediction (increment in *R*^2^ = 0.048, *F*(3, 24) = 2.09, MSE = 0.019, *p* = 0.12813). By contrast, the addition of the brain imaging measures to the psychometric measures resulted in a reliable improvement in prediction (increment in *R*^2^ = 0.47, *F*(4, 24) = 14.99, MSE = 0.007, *p* < 0.00001). Thus, including the brain imaging measures provides additional ability to predict comprehension in individual participants, and also provides additional insight into the psychological processes underlying the reading comprehension performance.

### Predicting passage difficulty from text properties

The prediction of passage-level comprehensibility based on brain activation measures was superior to the prediction based on readability measures of the texts. Coh-Metrix natural language processing procedures^62^ were used to describe each text on five measures of readability (Narrativity, Syntactic Simplicity, Word Concreteness, Referential Cohesion, and Deep Cohesion). (The inter-correlation of these measures is presented in Supplementary Table 2.) A stepwise regression using these predictors of individual passage comprehensibility indicated that only Syntactic Simplicity (which predicted better comprehension) approached significance in explaining the variance in passage comprehensibility (*R*^2^ = 0.24, *F*(1,14) = 4.45, MSE = 0.0001, *p* = 0.053386).

Although the Coh-Metrix measure of Deep Cohesion was not reliably related to passage difficulty, it was related to activation in two regions. As an alternative approach to explaining how features of the texts might influence comprehension processes, the Coh-Metrix measures were used to predict brain activation in each of the regions selected by the main stepwise regression relating activation to comprehensibility (L IFG L IPL, R TP, and M ACC/DMPFC). The stepwise regression predicting activation in L IFG found that only the measure of Deep Cohesion met the minimum Schwartz Bayesian Criterion (*R*^2^ = 0.45, *F*(1,14) = 11.51, MSE = 0.0001, *p* < 0.00438). The stepwise model for L IPL also selected only the measure of Deep Cohesion (*R*^2^ = 0.21, *F*(1,14) = 3.67, MSE = 0.0003, *p* = 0.076051). For the remaining two regions, stepwise regression models found no relationship between any of the six Coh-Metrix measures of text features and brain activation. Even though Deep Cohesion did not directly predict passage-level comprehensibility, it is possible that activation in L IFG and L IPL mediates the relationship between cohesion and comprehensibility. In both regions, more cohesive passages evoked greater activation, and greater activation in these regions was related to better comprehension of the passages.

### Predicting passage difficulty from familiarity with the content

Rated familiarity with the passage topics was only moderately related to comprehensibility. The passage topics were rated as relatively unfamiliar by the participants (mean rating = 2.83 (SD = 0.88) on a 7-point scale where 7 indicated “very familiar”). The mean of the correlations between a participant’s familiarity rating for a topic (based on having seen only the topic title) and their comprehension scores on the passage on that topic was modest (mean Fisher’s *z-*transformed *r* = 0.24, SD = 0.28). Note that each individual passage had a limited number of possible performance levels, based on responses to four multiple-choice test questions, each with four alternatives. This may limit the information these correlations can provide. Despite the weak correlation, this mean across participants was nevertheless reliably greater than zero (*t*(30) = 4.65, *p* < 0.000005).

### Changes in activation with repetitions of the passages

As expected, there were changes in activation over the course of the three readings of the passages, but they were not the simple monotonic changes one might predict if processes became more or less active with each additional exposure. These changes were evaluated using whole-brain paired *t*-tests among the three pairs of passage repetitions across all participants. The results indicated that overall activation increased between the first and second reading of the passages in the bilateral occipito-temporal cortex, in the left superior temporal sulcus and anterior middle temporal gyrus, and in the right temporal pole (see Supplementary Fig. 3a). These same areas decreased in activation from the second to third reading of the passages (see Supplementary Fig. 3b). In the right inferior frontal gyrus and the medial superior frontal gyrus, activation increased by the third reading. This inverted U-shaped function of activation in temporal language areas and the corresponding increase in lateral and medial frontal areas may reflect a shift from semantic to executive processing with repeated presentations.

## Discussion

This study produced two central findings concerning the processes involved in the comprehension of technical information from a text, namely: (1) it identified the neural and psychological processes that are related to individual differences in the comprehension of technical texts, and (2) it identified the neural and psychological processes that are related to more comprehensible versus less comprehensible passages.

Individuals with higher comprehension showed more activity during reading in the left inferior frontal lobe, the left superior parietal lobe, the bilateral dorsolateral prefrontal cortex, and the bilateral hippocampus. Processes subserved by these regions are consistent with the construction of mental models that incorporate spatial imagery and integrate prior semantic knowledge with new information. By contrast, participants with poorer comprehension had higher activation in the right inferior parietal lobe and the left and right ventromedial prefrontal cortex. Poorer comprehenders engaged in more processing of the meanings of individual words and more processing of episodic or autobiographical knowledge.

The comprehensibility of individual technical passages was associated with the difficulty of accessing the meaning elements of the concepts in the text and integrating the concepts with each other, a difficulty best predicted by the brain activity in anterior left temporal lobe areas, consistent with previous research on the role of these regions^37,38^. Better comprehended passages were also associated with more activity in an area of the right temporal lobe that is involved in establishing discourse level coherence across propositions, which is also consistent with previous research^63–66^.

The new insights into the neural and cognitive processes that underlie successful expository comprehension converge with prior behavioral research in their practical implications. These implications include suggestions for the teaching of particular comprehension strategies and for improving the writing of such texts to facilitate comprehension, as discussed in more detail in a later section.

Among the better comprehenders, there was more activation in the left inferior frontal gyrus, associated with phonological, semantic, syntactic, and propositional maintenance processes^67^ that constitute verbal working memory for language^60,68^. Individual differences in working memory capacity have long been known to predict language comprehension at multiple linguistic levels, including discourse-level comprehension^63,69^. The findings here show that more activation in this region during the reading of technical texts (enabling more maintenance of the text representation in an activated state) helps predict individual differences in comprehension performance.

Brain regions responsible for spatial processing and spatial imagery (SPL and IPS) were more active among better comprehenders, suggesting that their spatial cognition facilitated comprehension^70^. This was coupled with greater activation in lateral prefrontal semantic structure-building areas, suggesting that spatial imagery was used to construct an integrated mental model^71^. This is consistent with behavioral evidence that readers incorporate such spatial imagery into their situation models^72,73^. Spatial mental models may also serve as a medium for “mental animation” that can represent the causal and dynamic properties of a system^25^. The passages used here generally contained highly visuospatial conceptual content and the greater activation of the better comprehenders most likely reflects greater visualization of the content.

During the reading of the passages, better comprehenders showed greater activation in the bilateral anterior dorsolateral prefrontal cortex and bilateral hippocampus, whereas poorer comprehenders showed higher activation in the medial ventrolateral prefrontal cortex and the precuneus. The activation pattern in better comprehenders is associated with the encoding and integration of new semantic information with retrieved semantic knowledge to construct new knowledge structures^44,45^. By contrast, the activation in poorer comprehenders in more medial prefrontal areas is associated with relating new information to retrieved episodic memories or autobiographical knowledge^47–49^. Numerous psychological and educational studies have firmly established the role of prior knowledge in reading comprehension performance^7,8,30,35,74–76^. The implication of the present findings is that the comprehension of technical information is better when it is related to previous semantic knowledge than when it is related to previous episodic knowledge.

In summary, during the reading of expository texts, greater activation of regions associated with verbal working memory, spatial visualization, mental model construction, and the semantic integration of new and retrieved knowledge was observed in the better comprehenders.

Activation in frontal and temporal lobes that was related to individual passage difficulty reflects processes that integrate different types of semantic information from different cortical areas^56,57,77^. The right temporal pole’s activation in response to the more difficult passages may result from such texts containing information inconsistent with world knowledge. This region has previously been shown to activate during the comprehension of narrative texts that contain details that are inconsistent with a global theme^78^. In contrast, the right posterior temporal lobe activated more for passages that were better comprehended, consistent with its role in drawing inferences that are required to build a coherent situation model^63–66,78^. The current findings indicate that passage difficulty is modulated by processes that bring together information from anatomically distributed component representations and that bind together different types of information. The fMRI technology allows the underlying neural processes that occur during the actual reading to be observed and measured and subsequently related to the resulting comprehension performance.

The findings that better technical comprehension involves activation in regions associated with language and verbal working memory, spatial mental model construction, and knowledge integration support the teaching of several strategies that might improve comprehension of technical passages. Less familiar concepts could be introduced and explained prior to their mention in a text. This is consistent with educational research showing that skilled comprehenders have more immediate access to word meaning, facilitating the integration of individual words into a mental model^30^ and that teaching the meanings of unfamiliar concepts in the texts ahead of time improves text comprehension^79^. In addition, explicitly teaching visualization strategies may be an effective way to improve the performance of poorer comprehenders of technical material. Behavioral research has demonstrated that spatial ability is related to success in science, technology, engineering, and math (STEM) fields^80^, and that computer-based instruction in spatial visualization strategies improves performance in reading comprehension^81^. Explicitly teaching individuals to make the type of bridging and causal inferences that are necessary for building a situation model may also enhance the comprehension of technical passages. One of the most effective ways of facilitating such inference generation is teaching self-explanation^27,82–84^.

The finding that activation in areas associated with semantic access and integration was related to the difficulty of individual passages suggests that technical texts can be improved by making them more cohesive. A robust finding in the psychological and educational literature is that text cohesion facilitates coherence and improves comprehension of discourse^8,9,36,62,85^. Although the present study did not find reliable correlations between passage comprehension and measures of passage cohesion, the materials were not designed to vary greatly in cohesion. Nevertheless, the fMRI data seem to be sensitive to this aspect of the structure of the passages, as evidenced by the reliable correlations between a measure of deep cohesion and activation. This activation was predictive of comprehension and may serve as a mediating individual difference variable indicative of the interaction between the text and the brain in determining comprehension. Explicit cohesive devices that can be incorporated across sentences and paragraphs include overlap of arguments and clear indications of causal, temporal, and spatial relations that are not overtly stated.

This study represents an advance in understanding the sources of individual differences in comprehending complex technical information. The brain activation data showed that better comprehenders engage more of three key cognitive processes: they keep more verbal information temporarily active in memory while reading, they are more likely to imagine the spatial relationships among the objects described, and they relate new information with previously learned world knowledge. These findings suggest that teaching comprehension strategies that explicitly engage these processes could improve technical comprehension among students who are poorer at this task. In addition, passages that are harder to comprehend inadequately interrelate different types of information, evoking additional integrative and inferential processing. Instructional texts that make such relations more explicit may facilitate comprehension and learning.

As society becomes increasingly dependent on technical learning from text, the needs for new instructional approaches arise. Functional neuroimaging research can inform the quest for such meta-cognitive instructional approaches. The research on reading comprehension is just one example of the potential impact of this approach.

## Methods

### Participants

Thirty-one right-handed, native English speakers between the ages of 18 and 35 (25 females and 6 males) from the Pittsburgh area provided usable data in the fMRI scanning task. These participants were selected from a pool of 265 individuals who participated in an online reading comprehension test to identify participants at the extremes of this distribution of reading comprehension performance. Eleven participants were excluded from the fMRI analyses due to excessive head motion (8), because they fell asleep during the scan (2), or were found to have an anatomical brain abnormality (1). All participants gave signed informed consent approved by the Carnegie Mellon Institutional Review Board.

### Materials

Twenty-four passages (presented in Supplementary Table 3) were developed that described either mechanical devices or general knowledge topics. Each passage consisted of five sentences with a mean passage length of 132 words. The topics of the 16 passages used in the fMRI study were Bilge Pump, LiDAR, Refrigeration System, Automatic External Defibrillator, Screw Propeller, Sonar, 3D Printer, Aircraft Carrier Catapult, Bacteria, Acoustics and Cochlear Implants, Fever, Tumors Oncology Cancer, Photography, Intellectual Property, Beverages, and Mechanical Engineering of Robots. The eight remaining passages were used for pretesting of participants and familiarization with the reading comprehension task. The topics of the pretest passages were Boiler, Pressure Safety Valve, Turbine, Landing Gear Door Latching System, Automatic Direction Finder, N95 Masks, The Clock’s Timekeeper, and Electronic Circuits.

### Procedures

#### Pretest screening session

Prior to participation in the fMRI session, participants first completed two reading comprehension tasks in a one-hour interactive online Zoom session to identify those who were particularly good or particularly poor at technical comprehension. The participants read two iterations of the set of eight screening passages, presented as a 17-min video over Zoom. The presentation of screening passages followed the same procedures as the fMRI task, as described below. The fMRI study enrolled only participants with either high or low reading comprehension scores (High = 88–100% correct; Low = 41–72%) in the pretest. Following the comprehension test, the participants completed the Nelson–Denny Reading Comprehension Test^58^ as an additional assessment of reading comprehension skills.

#### Experimental session

Prior to the fMRI scanning, familiarity ratings on a 1–7-point scale for each of the 16 passage topics were acquired (1 being not familiar at all, and 7 being very familiar), along with a Handedness Questionnaire^86^.

Following the presentation of one warmup passage (on the topic of Wheelchairs), the set of 16 test passages was presented three times (in different random orders) by first displaying the passage title for 1.5 s, followed by a fixation point for 0.5 s. The passage was then displayed using a moving window paradigm that presented one phrase (consisting of 1–4 words) at a time. The segmentation into phrases attempted to respect syntactic boundaries. Phrases longer than 4 words were presented in two successive cumulating segments. The displayed phrase disappeared when the next phrase was presented. The moving window moved from left to right and down successive lines in the locations where the text would normally occur. To present the text at a comfortable reading rate, the presentation duration parameters were based on an approximate average modulation of gaze durations by word length and frequency during text reading^87^, plus an intercept of 300 ms. The phrase presentation duration was 300 ms + 16 ms per character + (400 ms–(31.26*log (word frequency of least frequent word))). There was a pause of 4 s after each of the first four sentences of a passage during which the fixation point (an asterisk) was displayed, providing time to process the most recent sentence as well as integrate the new information with the previous sentences. This echoes the natural pause observed at the ends of sentences^87^. After the final sentence, an ‘X’ was displayed for 6.5 s to mark the end of the passage.

During the first two presentations of the passages, the stems of two (of the ultimate 4) questions (but not the response alternatives) were presented following each passage, to orient the participants to the type of information that would be interrogated (see Supplementary Table 4). The same questions were presented in the first two presentations. The two questions were displayed simultaneously for 5–10 s (the precise duration being dependent on their character length). Following the presentation of the two questions, an ‘X’ fixation point, 3.5 s in duration, was presented before the next passage began.

After completing the comprehension task, 29 of the participants also performed a recall task in the scanner whose analysis is not reported here because of its marginal contribution to the goals of the main study.

In the post-scan comprehension test, comprehension was assessed with four multiple-choice questions (each having four possible response alternatives) regarding each of the 16 passages. The stems of two of the four questions per passage were presented in conjunction with the first and second presentations of the passages (see Supplementary Table 4). The other two questions had not been previously seen by the participants. Participants also completed the following psychometric tests after the scan: The Reading Span Test^60^, Raven’s Standard Progressive Matrices^88^, and the Bennett Mechanical Comprehension Test (abbreviated version)^61^.

### fMRI acquisition

Functional images were acquired on a Siemens Prisma (Erlangen, Germany) 3.0 T scanner at the Brain Imaging Data Generation & Education (BRIDGE) center, jointly operated by Carnegie Mellon University and the University of Pittsburgh. A multiband slice-accelerated BOLD spin-echo echo planar acquisition sequence^89^ was used to acquire 40, 3-mm-thick slices with no gap in each TR (1000 ms) using a multiband factor of 2, an AC-PC slice orientation covering all of the cerebral cortex, a TE of 25 ms, a tip angle of 64°, a 192 × 192 mm field of view and a 64 × 64 in-slice matrix. SPM software^90^ was used to correct head motion in the images and normalize them to the Montreal Neurological Institute template^91^.

### Data analysis

The mean percentage signal change (MPSC) relative to the fixation condition was computed at each gray matter voxel for each image of each stimulus presentation. The main measure of activation evoked by a passage consisted of the voxel activation levels acquired for each sentence around the peak of the hemodynamic BOLD response and averaged across the five sentences of the passage. The data acquisition interval for each sentence included four brain images acquired once per second (i.e. a TR of 1000) within a 4-s window, offset 5 s from the stimulus onset (i.e. images 5–8). This window was not dependent on the duration of the sentence presentation but rather was intended to capture the peak of the BOLD response to the sentence.

#### Identification of regions responsive to individual differences in comprehension

Cortical areas that responded differentially across participants during reading were identified by statistical parameter maps of voxelwise correlations between MPSC (averaged over all passages) and comprehension performance on the post-scan test. Voxelwise one-sample *t*-tests identified clusters of positive and negative correlations with comprehension. These analyses were performed in SPM using a height threshold of *p* < 0.05 and a cluster extent threshold of 10 voxels. A set of 12 regions of interest (ROIs) met these criteria for region selection (Fig. 1 and Table 1).

This ROI selection procedure was also performed separately for each of the three presentations (i.e. the three repeated readings of the set of 16 passages), as well as for each possible pair of presentations, to provide independent ROIs for uncontaminated cross-validation of the regression model.

#### Predictive modeling of individual differences in comprehension

The MPSC data (averaged over the three presentations) from the correlation-based ROIs were entered into a stepwise regression model as independent variables, with the participants’ mean comprehension scores (averaged over the four comprehension questions) for each passage as the dependent measure. The purpose of the stepwise analysis was to select from this full set of regions those that together contributed most to predicting comprehension performance. The stepwise selection procedure used a *p* < 0.15 criterion for entry and a *p* < 0.15 criterion for retention in the final model, which used the minimum Schwartz Bayesian Criterion for stopping. Multi-collinearity was assessed with variance inflation and tolerance measures at each step. To evaluate the fit of the resulting model (which included only MPSC data from the stepwise-selected ROIs), the predicted comprehension values from this model were compared to the observed comprehension values, resulting in an *R*^2^ value quantifying the variance in comprehension explained by the activation in the selected ROIs.

Cross-validation of the prediction of participant-level comprehension from participant-level activation was performed by training three separate models on the MPSC data from each possible pair of presentations of the passages, and by testing the resulting model by applying the obtained regression weights to the averaged MPSC data in the corresponding ROIs in the remaining, left-out presentation. ROIs in each training set were selected as the cluster of comprehension-correlated voxels whose peak was closest to that of the selected ROIs from the descriptive stepwise model above. The data from the test presentation was extracted from these training-based ROIs rather than those defined in the test presentations. The predicted comprehension performance was averaged for each participant over the three folds of the cross-validated models and was compared to the observed comprehension performance for that participant, resulting in a cross-validated *R*^2^ measure of the fit.

#### Identification of regions and predictive modeling of the comprehension of individual passages

Regions of interest (ROIs) for modeling the comprehensibility of individual passages were selected on the basis of statistical parameter maps of the voxelwise correlation between the mean comprehension of an individual passage (averaged over all participants) and the MPSC in each voxel. A one-sample *t*-test identified regions that were reliably positively or negatively related to comprehensibility over the 16 passages (threshold *p* < 0.05, minimum cluster size = 10). Thirteen ROIs met these criteria (Fig. 4 and Table 2).

This same ROI selection procedure was also performed separately for each of the three presentations (i.e. repeated readings of the set of 16 passages) to provide independent ROIs for uncontaminated cross-validation of the prediction of individual passage comprehension.

#### Multiple regression prediction of mean comprehension performance of each passage

Mean comprehensibility of individual passages was predicted using the same regression methods and criteria as those used for predicting individual participant comprehension. Stepwise selection was used to identify regions in which the MPSC significantly contributed to the prediction of the comprehensibility of individual passages, and the fit of the model was evaluated by the *R*^2^ value quantifying the variance in observed comprehensibility explained by the passage comprehensibility predicted by activation in the selected ROIs.

As with the prediction of participant-level comprehension performance, cross-validation of the ability of activation to predict passage-level comprehensibility involved fitting a separate model using the MPSC in the independently selected ROIs for each possible pair of presentations to predict comprehension performance and applying the resulting regression weights to the averaged activation in corresponding ROIs in the test presentation. The predicted comprehension performance was averaged for each individual passage over the three folds and was compared to the observed comprehension performance for that passage, resulting in a cross-validated *R*^2^ measure of the fit.

#### Assessing changes in activation across presentations

Correlation maps for each presentation based on participant-level and passage-level comprehension means were examined to qualitatively judge the consistency of the voxelwise relationships between comprehension and activation over the three presentations. To quantify changes in activation across presentations, the overall MPSC (the overall voxelwise mean signal during fixation subtracted from that during that passage reading) was calculated for each participant. Second-level voxelwise paired *t*-tests were then performed on this MPSC data for the 31 participants contrasting each pair of presentations. The resulting statistical parameter maps were thresholded (*p* < 0.05, minimum cluster size = 10), to identify regions that increased or decreased in overall comprehension activation relative to the baseline for each pair of presentations.

### Reporting summary

Further information on research design is available in the Nature Research Reporting Summary linked to this article.

### Supplementary information

Supplementary Information

Reporting summary

## Data Availability

All data are available by request directed to the corresponding author. There are no limits on data sharing.

## Code availability

All codes used for the analyses are available by request directed to the corresponding author.
