# Supplementary material for: The neural and cognitive basis of expository text comprehension
Source: NPJ Sci Learn. 2024 Mar 21;9:21. doi: 10.1038/s41539-024-00232-y (PMC10957871; doi:10.1038/s41539-024-00232-y)
Supplement: Supplementary file 1 — Supplementary Information [file 41539_2024_232_MOESM1_ESM.pdf]

Supplementary Information for:

The neural and cognitive basis of expository text comprehension

Timothy A. Keller[1]*, Robert A. Mason[1], Aliza E. Legg[1], & Marcel Adam Just[1]

This PDF file includes:

Supplementary Figures 1 to 4
Supplementary Tables 1 to 4

[1] Department of Psychology, Carnegie Mellon University, Pittsburgh, PA, USA.
* Corresponding author**.** e-mail: tk37@andrew.cmu.edu

SUPPLEMENTARY FIGURES

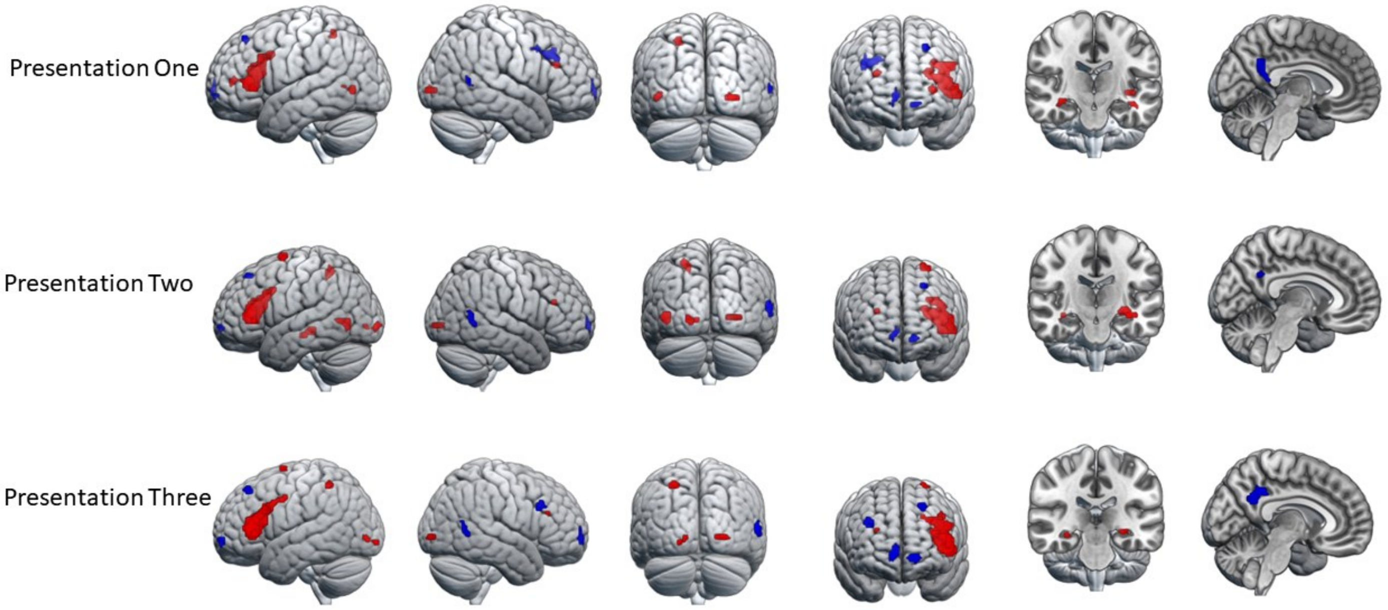

Supplementary Figure 1. Regions where brain activation was related to individual differences in reading comprehension in each presentation. Red: Regions where activation was greater for participants who were better comprehenders. Blue: Regions where activation was greater for participants who were poorer comprehenders.

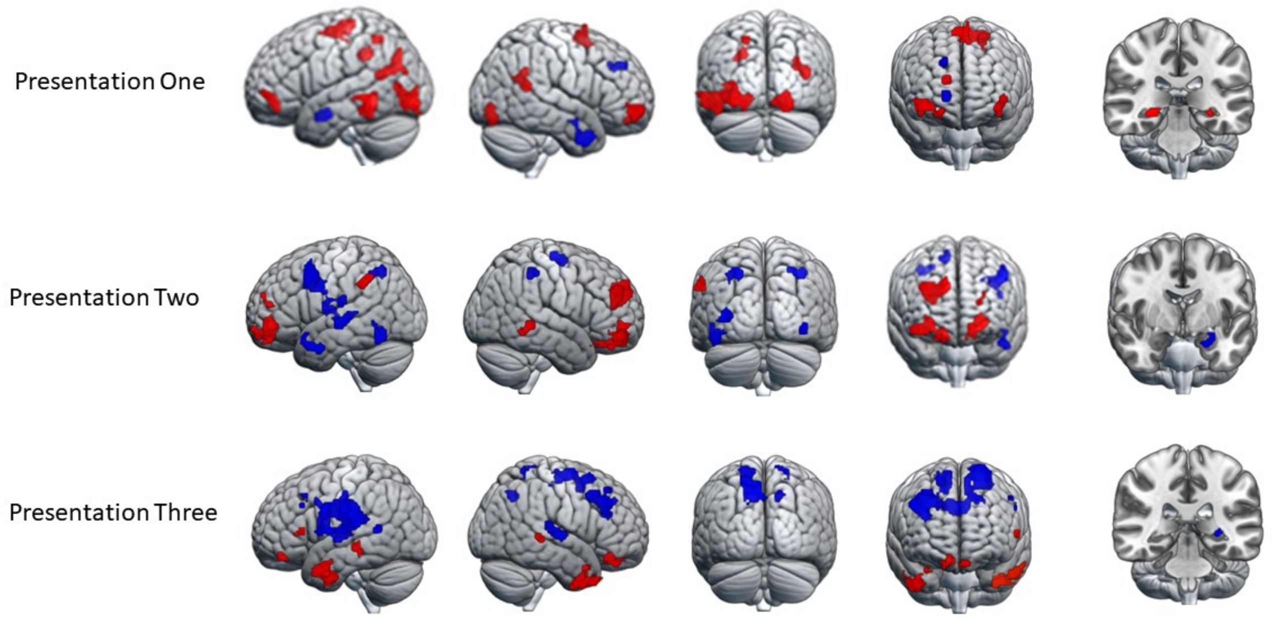

Supplementary Figure 2. Regions where brain activation was related to passage differences in reading comprehension in each presentation. Red: Regions where activation was greater for participants who were better comprehenders. Blue: Regions where activation was greater for participants who were poorer comprehenders.

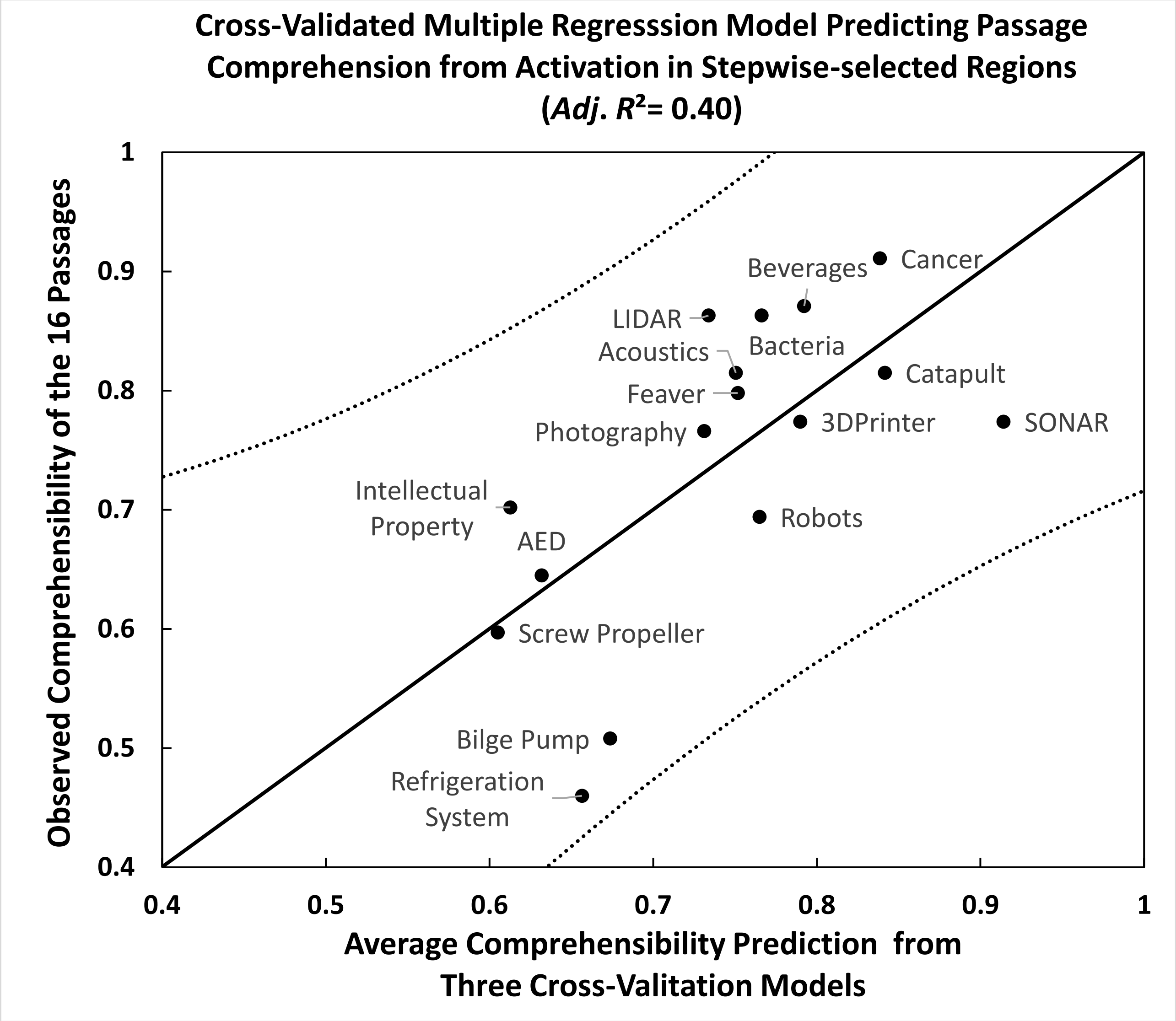

Supplementary Figure 3. Cross-validated relationship between individual passage comprehension performance and activation in key cortical regions. Each point represents a participant. Values on the axes are the proportion of correct responses on a multiple-choice comprehension test. Dotted lines are the 95% confidence intervals for the prediction. See Methods or Table S3 for the full passage titles.

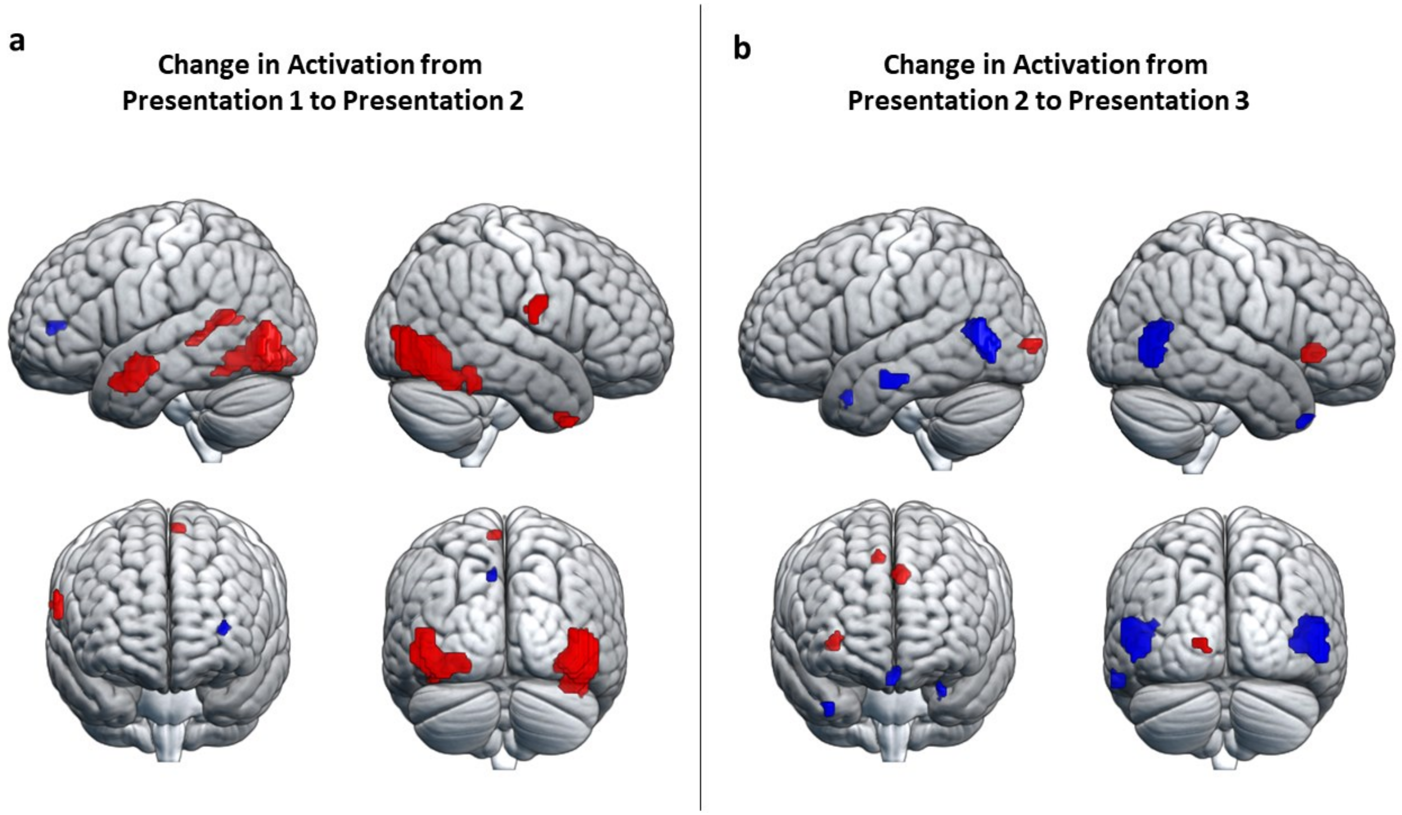

Supplementary Figure 4. Changes in brain activation across three readings of the passages. Red: Regions where activation increased. Blue: Regions where activation decreased.

SUPPLEMENTARY TABLES

Supplementary Table 1. Correlations among psychometric individual difference measures across all participants. Shaded cells are reliably correlated at $p < .05$.

	Post Scan Comprehension Test	Pre-screening Comprehension Test	Nelson-Denny Comprehension Score	Nelson-Denny Reading Rate	Daneman-Carpenter Reading Span	Ravens Progressive Matrices	Bennet Test of Mechanical Aptitude
Post Scan Comprehension Test	1						
Pre-screening Comprehension Test	0.76	1					
Nelson-Denny Comprehension Score	0.63	0.63	1				
Nelson-Denny Reading Rate	0.13	-0.12	-0.15	1			
Daneman-Carpenter Reading Span	0.47	0.48	0.37	0.03	1		
Ravens Progressive Matrices	0.54	0.48	0.36	0.04	0.53	1	
Bennet Test of Mechanical Aptitude	0.43	0.52	0.37	-0.31	0.36	0.61	1

Supplementary Table 2. Correlations among passage difference measures. The measures are z-scores of the Text Easability Principal Components obtained by submitting each of the 16 passages to the Coh-Metrix 3.0 tool. Shaded cells are reliably correlated at p <.05.

Post Scan Comprehension Test	1								
Narrativity	-0.25	1							
Syntactic Simplicity	0.49	-0.67	1						
Word Concreteness	-0.17	0.08	-0.27	1					
Referential Cohesion	-0.44	0.82	-0.74	-0.18	1				
Deep Cohesion	0.31	-0.62	0.71	-0.10	-0.68	1			
Verb Cohesion	-0.31	-0.24	-0.18	-0.12	0.16	-0.11	1		
Connectivity	-0.26	0.42	-0.65	-0.12	0.32	-0.57	0.09	1	
Temporality	-0.33	0.41	-0.51	0.18	0.63	-0.09	0.13	-0.10	1
	Post Scan Comprehension Test	Narrativity	Syntactic Simplicity	Word Concreteness	Referential Cohesion	Deep Cohesion	Verb Cohesion	Connectivity	Temporality

Supplementary Table 3. Sixteen expository technical passages presented in the experiment.

Topic	Passage
Bilge Pump	*The central part of a bilge pump, which pumps out standing water from the bottom of a ship, is an electrically powered impeller, which is like a rotary fan with curved, scoop like blades. The spinning of the impeller creates a low pressure in the impeller's center, which draws the standing water through an intake hose into the center of the impeller. As the impeller rotates, the centrifugal force (outward from center) causes the water to flow outward from the impeller's center and along its spinning blades. The water then flows from the ends of the blades into a surrounding set of channels, called diffusers. These surrounding diffuser channels gradually increase in size, decreasing the water's velocity and increasing its pressure, and this pressure forces the water out through a discharge port and out of the ship.*
LIDAR	*Self-driving cars rely on LiDAR (Light Detection and Ranging), an object-sensing method that uses pulsed laser light to measure the distance to surrounding objects. A LiDAR instrument on a self-driving car consists of a slowly spinning rooftop laser, a sensor to detect the reflected pulses, and an onboard computer. As the laser housing spins on top of the car, near-infrared laser pulses are sent out and then reflected back to the sensor by the surrounding objects. The onboard computer records each laser pulse's reflection point on the sensor, measures the time delay between each outgoing and reflected pulse, and determines the distance to the object based on the speed of light. The onboard computer then combines these distance measurements to generate a 3D model of the objects in the surrounding environment.*
Refrigeration System	*A refrigeration system cools a space by changing a fluid, called a refrigerant, from liquid to gas form in an evaporator, absorbing heat from the surrounding area. To turn the liquid refrigerant into a gas, the refrigerant is first put under high pressure and then allowed to flow through an expansion valve, which allows a drop in its pressure as it moves into the evaporator. The lowered pressure refrigerant then evaporates, taking in heat from the outside area as it changes from a liquid to a gas, and cooling off the area to be refrigerated. After the cooling occurs, the cycle starts again, with the gas moving into a compressor that compresses it, increasing the refrigerant's temperature and pressure. The now hot gas refrigerant flows into the condenser's long coils of tubing, where it releases heat to the outside air as it changes from a gas back into a liquid, and then flows through the expansion valve to the evaporator, completing the cooling cycle.*

Supplementary Table 3 (continued).

Topic	Passage
Automatic External Defibrillators	*Automatic External Defibrillators, AEDs, are small computerized devices that analyze heart rhythms and provide defibrillation, or the application of an electrical shock to stop arrhythmia of the heartbeat. AEDs include a battery, a device that stores electric charge called a capacitor, a speaker, sensor electrodes, and connector cables. The electrodes are first placed on the patient's chest to analyze the electrical activity of the heart and determine if the patient is in ventricular fibrillation and whether a shock is warranted. If a shock is warranted, the AED uses the battery to charge its capacitor and orally instructs the user to ensure no one is touching the patient before delivering the shock. The electrical charge then travels through the connector cables to the electrodes and to the heart, shocking the heart in an attempt to reestablish an effective heart rhythm.*
Screw Propeller	*Screw propellers are widely used to propel ships, from personal motorboats to battleships. On large ships, these propellers include a central hub called a boss, a number of radiating blades attached to the hub, and a propeller shaft running into the ship. Inside the ship, the ship's engine connects to and adds torque to the propeller shaft, rotating the shaft and central boss of the propeller. As the propeller's boss rotates, the attached curved blades move like a screw through the water, changing the rotational force of the propeller into a linear force, called thrust, by pushing the water away from the ship, which creates a thrust in the opposite direction. The resulting thrust moves from the propeller's boss, through the propeller shaft, to finally reach the hull of the ship and push the ship forwards.*
SONAR	*Sonar, or Sound Navigation Ranging, uses sound energy to detect objects on or under water. A sonar system sends out pulses of sound and listens for echoes, and this process uses a transmitter, receiver, and transducer. The transmitter creates an electric signal and sends it to the transducer, which then converts the electrical signal into a sound wave. The sound wave, called a 'ping', is then sent out into the water until it hits an object and bounces back as a returning echo, which hits the transducer and is converted back into an electrical signal. The length of time between transmitting the ping and receiving its echo is used to determine the distance between the object and the transmitter.*

Supplementary Table 3 (continued).

Topic	Passage
3D Printer	*A typical 3D printer is much like an inkjet printer but it prints multiple layers of filament material from the bottom upward until it builds up a 3D object. The main parts of the printer are a print bed, the filament material, and a heated print head with a nozzle. A computer-controlled motor moves the print head to define an object layer by layer. To print a layer, the plastic filament material is fed through the moving print head, which melts it and extrudes it out of the nozzle and onto the print bed. As each layer cools, it binds to the layer beneath it, combining several 2D layers into the final 3D object.*
Aircraft Carrier Catapult	*Because aircraft carriers have limited runway space, they use a catapult system to quickly accelerate planes for takeoff. The catapult system includes two pistons and cylinders below deck, and two metal bars called a towbar and a holdback above deck. The plane's front wheels attach to the towbar, the holdback is positioned on the back wheel to keep the plane in place, the catapult's pistons are locked in place, and the cylinders fill with high-pressure steam from the ship's reactors to build up pressure. The pilot brings the plane's engine to full power, and then the flight crew releases the pistons, which have enough force to cause the holdback to release and the plane to be launched. The steam pressure pulls the plane forward by the towbar, accelerating the plane until it reaches the end of the catapult and the towbar releases from the plane and allows takeoff from the aircraft carrier.*
Bacteria	*Microorganisms grow and multiply, creating colonies of bacteria that can live in either a symbiotic or parasitic relationship with other living organisms. Some bacteria form a symbiotic relationship with humans, contributing to the immune system and the process of digestion. Nearly all animal life is dependent on bacteria for survival because only bacteria can synthesize vitamin B12, which is involved in the metabolism of every cell in the human body. Other types of bacteria that are called pathogens form a parasitic relationship in which they feed on larger organisms and can cause diseases such as tetanus or interfere with host cell function. Pathogens can damage host cells directly by using them for nutrients or damage them indirectly by triggering an immune response that can harm the host cells.*

Supplementary Table 3 (continued).

Topic	Passage
Acoustics/ Cochlear Implants	*Acoustics includes the study of how sound is transmitted in an enclosed space. An echo is a reflection of some original sound, whereas a reverberation consists of several repeated echoes of a sound. The human ear cannot distinguish an echo from the original sound if the delay is less than 1/10 of a second, so an echo must return with enough delay in order to be perceived. Acoustics research on sound in the middle ear led to the creation of cochlear implants, which translate sounds into vibrations within the ear. The cochlear implant uses these vibrations to stimulate the auditory nerve in the inner ear, and create artificial hearing.*
Fever	*A fever, or pyrexia, is an increase in the body's temperature caused by illnesses, including viral, bacterial, and parasitic infections. During an infection, chemicals called pyrogens enter the bloodstream and make their way to the hypothalamus in the brain, which regulates body temperature. When pyrogens bind to certain receptors in the hypothalamus, body temperature increases. Fevers need to be monitored because the level of fever indicates the severity of the illness, and any sudden increase in fever indicates a worsening of an illness. Certain types of medications called antipyretics can lower a fever, and if the temperature increases to dangerous levels, ice baths can be used to lower the body temperature.*
Tumors/ Oncology/ Cancer	*Cancer, the presence of malignant tumors, is the name for a group of diseases involving abnormal cell growth with the potential to migrate from one part of the body to other parts. Unlike cancerous tumors, benign tumors do not spread to other parts of the body, although they may still produce negative health outcomes. One form of radiation therapy for cancer uses ionizing radiation to kill cancer cells and shrink tumors, and its effects are localized and confined to the irradiated region. Two separate beams of radiation transmitted at different angles intersect precisely at the tumor site, sparing surrounding tissue from the radiation of the two beams. Although radiation can damage both cancer and normal cells, most normal cells can recover.*

Supplementary Table 3 (continued).

Topic	Passage
Photography	*Photography is the art of creating images with a camera that records patterned light either electronically when it falls on a sensor or chemically when it falls on film. The darkness of darks and lightness of lights in a photograph depends on exposure, which is the amount of light that hits the camera's sensor or light-sensitive film. Proper exposure is a balance between aperture size (the size of the opening that lets light in through the lens) and shutter speed (the amount of time the aperture is left open to allow light through). A slow shutter speed leaves the aperture open longer and can produce a blurred image of a fast-moving object. A slow shutter speed can also allow enough light from a poorly illuminated scene (such as a night scene) to accumulate and be photographed.*
Intellectual Property	*Intellectual properties are creations of human intellect, and different types of intellectual properties are protected by different types of legal rights including copyrights, patents, and trademarks. Copyrights apply to a wide range of artistic forms, but are mainly for books and music recordings, giving authors the distribution rights for a limited time. Patents protect the use of inventions or technological improvements, while a trademark protects the use of a logo or design. A royalty is a payment made to the owner of an intellectual property as specified by a licensing agreement.*
Beverages	*Beverages, which provide essential fluids for life and play a significant social role in human culture, are created using several different processes. Infusion, which can be traced to ancient times, is used for brewing teas by extracting flavors from plant material and allowing the material to remain suspended within water. Modern teas can also include additional steps such as flavoring, decaffeination, and mixing with sugar and milk, depending on taste preference. Fermentation, a metabolic process that converts sugar to ethanol, is used for making wine and brewing beer. To produce liquor, fermentation is followed by distillation, which boils the liquid and evaporates away components that have a lower boiling point, increasing the alcohol concentration.*

Supplementary Table 3 (continued).

Topic	Passage
Robotic Engineering	*Robotics involves the mechanical engineering of various types of robots that can differ in design, construction, operation, and purpose. All types of robots include some kind of mechanical system to enable movement, an electrical component for power, and computer programs to control the robot's actions. Spot, a four-legged robotic dog, is powered by electric motors and hydraulic pumps, and was designed to climb stairs and trot over rough terrain. Topio, a humanoid robot, was designed to play table tennis and learn new table tennis strategies using an advanced artificial intelligence system. Advances in artificial intelligence have also enabled robots to recognize speech, gestures, and facial expressions.*

Supplementary Table 4. Multiple-Choice Comprehension Test Administered After the Scanning Session. Correct responses are underlined. The first two questions listed were presented in the scanner following the corresponding passage during each of the first and second passage readings, but the response alternatives were not. The remaining two questions (in bold here) were not presented during the scanning and were new to the participant at the time of the test.

Topic: Bilge Pump

1. Standing water is sucked into a bilge pump's intake hose because of:
    a. The diffuser channels' increasing size.
    b. The low pressure at the center of the impeller.
    c. The decreased velocity of the standing water.
    d. The high pressure at the discharge port.

2. As the water flows through the diffuser channels that increase in size, its velocity decreases and pressure increases, causing it to:
    a. Reverse its direction of flow.
    b. Resist the centrifugal force.
    c. Move towards the center of the impeller.
    d. Flow out through the discharge port.

3. **The ______________ causes the water to flow outward from the impeller's center and along its spinning blades.**
    a. Diffuser.
    b. Intake hose.
    c. Centrifugal force.
    d. Discharge port.

4. **What part of the bilge pump is responsible for pumping out standing water from the bottom of the ship?**
    a. The external pressure.
    b. The center of the impeller.
    c. The intake hose.
    d. The electrically powered impeller.

Topic: LiDAR

1. Which of the following is a function of the LiDAR's onboard computer?
    a. It measures the weight of the surrounding vehicles.
    b. It generates a 3D model of the environment.
    c. It has advanced capabilities to automatically stop a vehicle to avoid an accident.
    d. It acts as a GPS and can select the best route based on the current traffic and accidents present.

2. What is the function of a self-driving car's LiDAR system?
    a. It uses video images to determine the distance between the car and the objects in its surroundings.
    b. It analyzes the flow of traffic within a certain area.
    c. It uses reflected laser pulses to determine the distance between the car and the objects in its surroundings.
    d. It communicates and coordinates its car's movements with surrounding cars.

Supplementary Table 4 (continued).

3. **What is used to measure the distance to the surrounding object?**
    a. **A satellite area reader.**
    b. **Pulsed laser light.**
    c. **Scope pulse sequence.**
    d. **A circumference detector.**

4. **What are the three components that make up the LiDAR instrument of a self-driving car?**
    a. **A laser, a sensor, and a computer.**
    b. **A light source, a 3D printer, and an AI system.**
    c. **A satellite, a pulse meter, and a mirror.**
    d. **A computer, a detectable signal, and the speed of light.**

Topic: Refrigeration System

1. When in the refrigeration cycle does the cooling actually occur?
    a. When refrigerant is compressed, increasing its pressure.
    b. When refrigerant flows through the condenser's coils of tubing.
    c. When refrigerant is changed into a gas, absorbing heat.
    d. When refrigerant is expanded, raising its pressure.

2. Which of these is a part of the refrigeration system?
    a. Condenser.
    b. Rectifier.
    c. Accelerator.
    d. Radiator.

3. **What happens when the gas changes into a liquid?**
    a. **The liquid refrigerant evaporates, taking in heat from the outside area as it changes from a gas to a liquid, and cooling off the area to be refrigerated.**
    b. **The liquid triggers the cycle to start again by flowing into a compressor and increasing the temperature.**
    c. **The liquid flows into the condenser's long coils of tubing and releases heat to the outside air.**
    d. **The liquid flows to the expansion valve to the evaporator and the cooling cycle is complete.**

4. **What is the first step in the refrigeration system process?**
    a. **The lowered-pressure refrigerant evaporates, taking in heat from the outside area as it changes from a liquid to a gas.**
    b. **The liquid refrigerant turns into a gas by being put under high pressure.**
    c. **The refrigerant flows through an expansion valve, which allows a drop in its pressure as it moves into the evaporator.**
    d. **Gas moves into a compressor that compresses the refrigerant, increasing the refrigerant's temperature and pressure.**

Supplementary Table 4 (continued).

Topic: Automatic External Defibrillator (AED):

1. What is the first function that an AED performs?
   a. The AED's battery charges the capacitor.
   b. The AED delivers a shock to the patient's heart.
   c. The AED instructs the user to ensure no one is touching the patient.
   d. <u>The AED analyzes the patient's heart rhythm.</u>

2. Which scenario would be the clearest indicator for the use of an AED?
   a. When a person is having difficulty breathing.
   b. When a person's heart has stopped beating.
   c. <u>When a person is in ventricular fibrillation.</u>
   d. When a person has lost consciousness.

3. **What is the piece of equipment that stores electric charge in an AED?**
   a. A battery.
   b. An EKG.
   c. An electrical sensor.
   d. <u>A capacitor.</u>

4. **What five pieces of equipment that are included in an AED device?**
   a. A shock meter, an EKG, a heart monitor, connecting coils, and electrical sensors.
   b. <u>A battery, a capacitor, a speaker, sensor electrodes, and connector cables.</u>
   c. A power cord, a discharge port, a control panel, connecting wires, and an outlet.
   d. A ventricular fibrillatory device, heart rhythm monitor, power strip, electrical connectors, and a respiratory enhancer.

Topic: Screw Propeller

1. What change in force is produced by a Screw Propeller?
   a. It changes the ship's linear force into rotational force.
   b. <u>It changes the propeller's rotational force into linear force.</u>
   c. It changes the ship's backward force into forward force.
   d. It provides a mechanism for steering the ship.

2. Which of the following statements is true about the capabilities of a Screw Propeller?
   a. A large ship that uses a screw propeller is called a boss.
   b. The forward motion of the ship causes the rotation of the propeller shaft.
   c. <u>The blades of the propeller push water away from the ship</u>.
   d. The thrust motion of water moving away from the ship causes the rotation of the propeller shaft.

3. **Inside the ship, the ship's engine connects to and adds ___________, which results in rotating the shaft and the central boss of the propeller.**
   a. Acceleration to the propeller's boss.
   b. Thrust to the propeller blades.
   c. <u>Torque to the propeller shaft.</u>
   d. Velocity to the discharge port.

Supplementary Table 4 (continued).

4. **What is the main goal of a screw propeller?**
   a. **To pump out standing water from the center of the ship.**
   b. **To rotate the ship in a clockwise motion.**
   c. **<u>To propel the ship forward.</u>**
   d. **To provide electrical energy to the engine of the ship.**

Topic: SONAR

1. How does sonar calculate the distance of an object?
   a. The decrease in the size between the transmitted ping and its echo indicates the distance.
   b. The decrease in the frequency between the transmitted ping and its echo indicates the distance.
   c. The number of returning echoes compared to the number of transmitted pings indicates the distance.
   d. <u>The length of time between transmitting the ping and receiving its return echo indicates the distance.</u>

2. What does the transducer in a sonar system do?
   a. <u>It converts electrical signals into sound waves and vice versa.</u>
   b. It measures the difference in the amplitude between the outgoing and returning sound wave.
   c. It travels outwards until it hits an object.
   d. It creates an echo of the outgoing sound wave.

3. **A sonar system sends out pulses of sound and listens for echoes, and this process uses:**
   a. **A satellite, sound wave and transductor.**
   b. **<u>A transmitter, receiver, and transducer.</u>**
   c. **A conductor, battery, and echo signal.**
   d. **An electrical signal, pulse meter, and radar.**

4. **What is another name for the sound wave?**
   a. **An echo.**
   b. **A signal.**
   c. **<u>A ping.</u>**
   d. **A vibration.**

Topic: 3D Printer

1. How are the 2D layers combined to form a 3D structure?
   a. The nozzle sprays an adhesive to bind adjoining layers to each other.
   b. The layers of plastic filament material are fused together using a wire mesh.
   c. <u>The adjacent heated layers of filament material fuse together as they cool.</u>
   d. Ultraviolet light heats and bonds adjacent filament layers after they are printed.

2. Which is true about 3D printers in terms of the direction that the object is printed?
   a. The object is printed left to right.
   b. The object is printed top to bottom.
   c. The object is printed right to left.
   d. <u>The object is printed bottom to top.</u>

Supplementary Table 4 (continued).

3. **What component of the 3D printer is responsible for moving the print head to define an object layer by layer?**
    a. **<u>A computer-controlled motor.</u>**
    b. **An electrical battery.**
    c. **A heated print head.**
    d. **A digital monitor.**

4. **What material is needed for a 3D printer?**
    a. **Fiber cloth.**
    b. **Rubber.**
    c. **<u>Plastic.</u>**
    d. **Paper.**

Topic: Aircraft Carrier Catapult

1. How is an aircraft carrier catapult used?
    a. Instead of using a plane's engine, it is used to accelerate a plane for takeoff.
    b. Instead of using a plane's brakes, it is used to decelerate a plane during landing.
    c. <u>Along with using a plane's engine, it is used to accelerate a plane for takeoff.</u>
    d. Along with using a plane's brakes, it is used to decelerate a plane during landing.

2. Which of these mechanisms does an aircraft carrier catapult use?
    a. <u>High-pressure steam powers a towbar that pulls the plane forward.</u>
    b. A powerful electric motor pushes the plane along the runway.
    c. A turbine adds lift to the plane's wings for takeoff.
    d. A belt system accelerates the plane's wheels down the runway.

3. **Once the pilot brings the plane's engine to full power, the flight crew releases ____________ which ______________________________________**
    a. **<u>The pistons; have enough force to cause the holdback to release and the plane to be launched.</u>**
    b. **The catapult system; quickly accelerates the planes for takeoff.**
    c. **The holdback; keep the plane in place and allow pressure to build up.**
    d. **The hydraulic cylinders; fill with high-pressure steam from the plane's reactors to build up pressure.**

4. **What is the main reason why aircraft carriers use a catapult system to quickly accelerate planes for takeoff?**
    a. **The length of the wings of the aircraft.**
    b. **The activation of the hydraulic system.**
    c. **The weight of an aircraft.**
    d. **<u>Limited runway space.</u>**

Supplementary Table 4 (continued).

Topic: Bacteria

1. Which statement is true about the function of bacteria?
    a. Bacteria do not form relationships with pathogens.
    b. Bacteria in symbiotic relationships damage host cell functioning.
    c. Bacteria are always detrimental to other organisms.
    d. <u>Bacteria are essential for the synthesis of some life-sustaining vitamins.</u>

2. Which is a way that pathogens damage host cells?
    a. <u>They trigger an immune response that damages host cells.</u>
    b. They trigger an increase in activity of the digestive system.
    c. They destroy vitamin B12 in host cells.
    d. Pathogens never damage host cells and are vital for survival.

3. **What type of relationship do bacteria form with other living organisms that contribute to the immune systems and the process of digestion?**
    a. A parasitic relationship.
    b. A positive relationship.
    c. <u>A symbiotic relationship.</u>
    d. A mutualistic relationship.

4. **Other types of bacteria that are called pathogens form a _____ relationship in which they ______**
    a. <u>Parasitic; feed on larger organisms and can cause diseases such as tetanus or interfere with host cell function.</u>
    b. Symbiotic; contribute to the immune system and the process of digestion.
    c. Negative; decrease the protection level of the organism that is naturally built up over time.
    d. Mutualistic; provide both helpful elements of protection against viruses and harmful introductions of bacterial and fungal colonies of microorganisms.

Topic: Acoustics and Cochlear Implants

1. What is the difference between an echo and a reverberation?
    a. A reverberation is a reflection of some original sound, while an echo consists of several repeated reverberations of a sound.
    b. <u>An echo is a reflection of some original sound, while reverberation consists of several repeated echoes of a sound.</u>
    c. An echo can only be heard from a short distance away, while reverberation can be heard much farther away.
    d. Reverberation can only be heard from a short distance away, while an echo can be heard much farther away.

2. How does a cochlear implant work?
    a. It dampens echoes within the middle ear to make it easier for the acoustic signal to reach the auditory nerve.
    b. It lengthens the time between echoes to make them easier to reach the auditory nerve.
    c. It repeats sounds using reverberation to make the acoustic signal easier to hear.
    d. <u>It translates sounds into vibrations within the ear, which are then used to stimulate the auditory nerve.</u>

Supplementary Table 4 (continued).

3. ______________ research on sound in the ____________ led to the creation of cochlear implants.
   a. Reverberation; cochlea.
   b. Sound wave; ear canal.
   c. <u>Acoustics; middle ear.</u>
   d. Echo; inner ear.

4. What is stimulated in the inner ear to create artificial hearing?
   a. <u>Auditory nerve.</u>
   b. Vestibular nerve.
   c. Cochlea.
   d. Auditory tube.

Topic: Fever

1. Which is true about the definition of pyrogens?
   a. They are medications that can cause a fever.
   b. They are indicators of the end of a fever.
   c. They are eliminated by ice baths.
   d. <u>They are chemicals that interact with the hypothalamus.</u>

2. What does a sudden increase in fever indicate?
   a. <u>The onset of an infection.</u>
   b. The onset of bacterial illness.
   c. The onset of a viral illness.
   d. The onset of a parasitic illness.

3. What are antipyretics?
   a. Antibiotics.
   b. Antifungal.
   c. <u>Medications used to lower a fever.</u>
   d. What binds to a receptor in the hypothalamus.

4. What happens in the body when someone has a fever?
   a. <u>A pyrogen binds to a receptor in the hypothalamus</u>
   b. A pyrogen is released from the hypothalamus
   c. An antipyretic is released from white blood cells as an immune response
   d. An antipyretic enters the bloodstream and raises the body temperature

Topic: Tumors/Oncology/Cancer

1. Which one of the following statements accurately characterizes the definition of radiation therapy?
   a. It damages body parts that are far from the tumor.
   b. <u>It uses ionizing radiation to kill cancer cells.</u>
   c. It damages only cancer cells.
   d. It is used to treat benign tumors.

Supplementary Table 4 (continued).

2. How does cancer radiation therapy avoid damaging tissues that are far from the tumor?
    a. The ionizing radiation energy is weakened after it passes through the tumor.
    b. The radiation is programmed to remain longer in the tumor than in surrounding tissues.
    c. <u>The radiation is emitted as two separate beams that cross each other only at the tumor</u>.
    d. Lead shields placed over the patient's body expose only the tumor area to the radiation.

3. **What type of therapy was discussed as a potential treatment for cancerous tumors?**
    a. **Chemotherapy.**
    b. **Radioactive therapy.**
    c. **Psychodynamic therapy.**
    d. **<u>Radiation therapy.</u>**

4. **What is the purpose of ionizing radiation?**
    a. **To relocate tumors.**
    b. **To surgically remove tumors.**
    c. **<u>To kill cancer cells and shrink tumors.</u>**
    d. **To promote normal cell growth around the tumor location.**

Topic: Photography

1. What is shutter speed?
    a. The amount of time taken by film to chemically react to light.
    b. The amount of time used for a camera flash.
    c. <u>The amount of time the aperture is left open to allow light into camera.</u>
    d. The amount of time the camera's sensor is turned on.

2. Which of the following is true about the balance of exposure in photography?
    a. Proper exposure is a balance between shutter speed and focus.
    b. Proper exposure is a balance between aperture size and focus.
    c. Proper exposure is a balance between focus and external lighting.
    d. <u>Proper exposure is a balance between aperture size and shutter speed.</u>

3. **What is aperture?**
    a. **<u>The size of the opening that lets light in through the lens</u>.**
    b. **The amount of time that is left open to allow light through.**
    c. **The amount of light that hits the camera's sensor or light-sensitive film.**
    d. **The balance between exposure and shutter speed.**

4. **What can occur when aperture is open longer?**
    a. **A night scene.**
    b. **A black and white image.**
    c. **A very bright image.**
    d. **<u>A blurred image.</u>**

Supplementary Table 4 (continued).

Topic: Intellectual Property

1. Which of these definitions about intellectual property is correct?
    a. Patents protect a logo or design.
    b. Trademarks protect books and music recordings.
    c. <u>Patents protect the use of technological improvements.</u>
    d. Copyrights protect a logo or design.

2. Which of these is a type of intellectual property legal right?
    a. <u>Patent.</u>
    b. Royalty.
    c. Equity.
    d. License.

3. **What is a payment made to the owner of an intellectual property?**
    a. **Patent.**
    b. **<u>Royalty.</u>**
    c. **Equity.**
    d. **Copyright.**

4. **What does the licensor have the right to of their intellectual property?**
    a. **Any profit from using the intellectual property.**
    b. **<u>Receiving a percentage of the profit as the royalty.</u>**
    c. **The patent of the property.**
    d. **The distribution rights of the property.**

Topic: Beverages

1. What is fermentation?
    a. <u>A metabolic process that converts sugar to ethanol.</u>
    b. The process of extracting flavors from plant material and allowing it to suspend in water.
    c. A selective boiling process to separate mixtures.
    d. A process that can include additional steps such as flavoring and decaffeination.

2. Which beverage-making process is used to make tea?
    a. Distillation.
    b. <u>Infusion.</u>
    c. Fermentation.
    d. Malting.

3. **What beverage making process has been traced back to ancient times?**
    a. **Distillation.**
    b. **<u>Infusion.</u>**
    c. **Fermentation.**
    d. **Malting**

Supplementary Table 4 (continued).

4. _________ is followed by __________ to produce liquor.
   - **a. Decaffeination; flavoring.**
   - **b. Converting sugar; converting ethanol.**
   - **c. Infusion; malting.**
   - **d. <u>Fermentation; Distillation.</u>**

Topic: Mechanical Engineering of Robots

1. The defining characteristic of a modern robot is:
   - a. The ability to recognize spoken language.
   - b. <u>The combination of physical action and artificial intelligence.</u>
   - c. Having an electrical motor that enables complex actions.
   - d. Having the general look of an animate being with limbs that move.

2. What are two of the components that are needed in order to enable the movement of a robot?
   - a. A chemical and an electrical component.
   - b. A physical and a theoretical component.
   - c. <u>A computer program and a mechanical component.</u>
   - d. An electrical component and a power component.

3. What allowed a humanoid robot to play table tennis and learn new table tennis strategies?
   - **a. <u>An advanced artificial intelligence system.</u>**
   - **b. A mechanical system that enabled complex movement.**
   - **c. A powerful electrical motor and hydraulic pumps.**
   - **d. A computer program and physical components.**

4. In order to qualify as a robot, a device has to be able to:
   - **a. have the same shape as a human being**
   - **b. <u>take physical actions that are guided by intelligence</u>**
   - **c. move and function within some physical environment**
   - **d. traverse similar types of terrains that humans can traverse**
